# Suicide Trends during the COVID-19 pandemic and the International COVID-19 Suicide Prevention Research Collaboration: Ann John

**DOI:** 10.1093/eurpub/ckac129.527

**Published:** 2022-10-25

**Authors:** A John, J Pirkis, D Gunnell, M Spittal, M Del Pozo Banos, V Arya, S Shin

**Affiliations:** 1 Swansea University Medical School, Swansea University, Swansea, UK; 2 Centre for Mental Health, University of Melbourne, Melbourne, Australia; 3 National Institute for Health and Care Research Bristol Biomedical Research Centre, University of Bristol, Bristol, UK

## Abstract

**Introduction:**

There was and still is much speculation about the COVID-19 pandemic impact on suicide rates. We aimed to assess the effect of the COVID-19 pandemic on suicide rates around the world.

**Methods:**

We sourced real-time suicide data from countries or countries areas through a systematic internet search (official websites of Ministries of health, police agencies, and government-run statistics agencies or equivalents), recourse to our networks (e.g. ICSPRC) and the published literature (a living systematic review). We used an interrupted time-series analysis to model the trend in monthly suicides before COVID-19 in each country or country area, comparing the expected number of suicides derived from the model with the observed number of suicides in the early months of the pandemic (from April 1 to July 31, 2020, in the primary analysis). We have now updated this work to cover the first 15 months of the pandemic and stratified analyses by age and sex and method. We will present findings from the new updated data (35 countries) at the conference.

**Results:**

Initially we sourced data from 21 countries (16 high-income and five upper-middle-income countries). Rate ratios (RRs) and 95% CIs based on the observed versus expected numbers of suicides showed no evidence of a significant increase in risk of suicide since the pandemic began in any country or area. There was statistical evidence of a decrease in suicide compared with the expected number in 12 countries or areas.

**Conclusions:**

This was the first study to examine suicides occurring in the context of the COVID-19 pandemic in multiple countries. Early on high-income and upper-middle-income countries, suicide numbers remained largely unchanged or declined compared with the expected levels based on the pre-pandemic period. We need to remain vigilant and be poised to respond as the longer-term mental health and economic effects of the pandemic unfold. We will present updated findings with more recent data.

